# Use of eHealth by Patients With Rheumatoid Arthritis: Observational, Cross-sectional, Multicenter Study

**DOI:** 10.2196/19998

**Published:** 2021-01-29

**Authors:** Marion Magnol, Berard Eleonore, Rempenault Claire, Benjamin Castagne, Marine Pugibet, Cédric Lukas, Anne Tournadre, Pascale Vergne-Salle, Thomas Barnetche, Marie-Elise Truchetet, Adeline Ruyssen-Witrand

**Affiliations:** 1 Rheumatology Department Toulouse University Hospital Toulouse France; 2 Rheumatology Department Bordeaux University Hospital Bordeaux France; 3 Rheumatology Department Montpellier University Hospital Montpellier France; 4 Rheumatology Department Le-Puy-En-Velay Hospital Le-Puy-En-Velay France; 5 Rheumatology Department Limoges University Hospital Limoges France; 6 Rheumatology Department Clermont-Ferrand University Hospital Clermont-Ferrand France

**Keywords:** eHealth, internet, mobile app, rheumatoid arthritis, patients’ expectation

## Abstract

**Background:**

The use of eHealth tools (eg, the internet, mobile apps, and connected devices) in the management of chronic diseases and for rheumatoid arthritis is growing. eHealth may improve the overall quality of care provided to patients with chronic diseases.

**Objective:**

The primary objective of this study was to describe eHealth use by patients with rheumatoid arthritis in France. The secondary objectives were to identify associations between patient demographics and disease characteristics and the use of eHealth tools, and assess their expectations of eHealth.

**Methods:**

In this cross-sectional, multicenter study, patients with rheumatoid arthritis, according to the 2010 ACR/EULAR classification criteria, were recruited from 5 university hospitals (Bordeaux, Clermont-Ferrand, Limoges, Montpellier, and Toulouse). Patients completed an anonymous self-questionnaire, including demographic data, evaluating their eHealth use (ie, access, support, frequency of use, type of use, and reason for use). The rheumatologist in charge of each patient completed an independent medical questionnaire on disease characteristics, activity of rheumatoid arthritis, and treatments. Data were collected between December 2018 and July 2019.

**Results:**

Questionnaires were completed by 575 participants, with a mean age of 62 (SD 13) years, 447 (77.7%) of whom were female. Overall, 82.2% (473/575) of the participants had access to eHealth through a computer (402/467, 86.1%), tablet (188/467, 40.2%), or smartphone (221/467, 47.3%). Of these, 36.4% (170/467) of the participants used the internet for health in general, and 28.7% (134/467) used it specifically for rheumatoid arthritis–related reasons. All these 134 patients used eHealth to learn about disease pathology, and 66.4% (89/134) of them used it as a tool to help monitor rheumatoid arthritis. Most patients (87/125, 69.6%) had a paper file, 19.2% (24/125) used a digital tool (spreadsheets, 10/125, 8%; mobile app, 9/125, 7.2%; or website, 5/125, 4%), and 24.8% (31/125) did not use any tools for monitoring. Few patients (16/125, 12.8%) used tools for treatment reminders. About 21.6% (27/125) of the patients using eHealth used a specific app for rheumatoid arthritis. Univariate analysis showed that age, education level, employment status, treatment, comorbidities, membership of a patient association, and patient education program were associated with eHealth use for rheumatoid arthritis. Multivariate analysis showed that membership of a patient association (odds ratio [OR] 5.8, 95% CI 3.0-11.2), use of biologic disease-modifying antirheumatic drugs (OR 0.6, 95% CI 0.4-1.0), and comorbidities (OR 0.7, 95% CI 0.6-0.8) remained associated with eHealth use for rheumatoid arthritis. Recommendation by a doctor (225/330, 68.2%), ease of use (105/330, 31.8%), and data security (69/330, 20.9%) were factors favoring the use of eHealth.

**Conclusions:**

To date, few patients have used eHealth for disease management. The use of a reliable and validated eHealth tool for rheumatoid arthritis could therefore be promoted by rheumatologists and could optimize therapeutic adherence.

## Introduction

Rheumatoid arthritis (RA) is one of the most common chronic autoimmune inflammatory rheumatic diseases with a prevalence of 0.1%-1% [1]. The disease is primarily characterized by a history of painful and swollen joints leading to joint deformation and destruction and disability. Nowadays, a wide range of disease-modifying antirheumatic drugs (DMARDs) are available (eg, synthetic, biologic, and targeted synthetic DMARDs). Because RA is a chronic disease, DMARDs are long-term maintenance treatments used to control the activity of rheumatism and prevent further joint destruction and disability.

In other chronic diseases such as diabetes, asthma, and hypertension, the use of eHealth has been well established. eHealth could be defined as an overreaching term used to describe the application of information and communication technologies in the health sector [2]. Studies show that eHealth and eHealth tools (ie, mobile apps, internet-based software and websites, connected devices, and personal health records) are ways to enhance medication adherence and disease control [3-5]. These eHealth tools provide information about the disease and help patients to monitor and manage the disease by improving patient autonomy.

Several websites, mobile apps, and connected devices related to RA have been developed for patients to obtain information about, self-monitor, or self-manage the disease. However, the impact of these specific eHealth services on disease management, medication adherence, or quality of life has been poorly assessed in the literature [6-9].

To date, there are no studies investigating the use of eHealth tools by patients with RA in France. Therefore, the main objective of this study was to describe the use of eHealth by patients with RA in France. The secondary objectives were to identify associations between patient demographics and disease characteristics and the use of eHealth tools, as well as to assess patients’ expectations of eHealth.

## Methods

### Study Design

A cross-sectional, multicenter, observational study was conducted in the rheumatology departments of 5 university hospitals in France (located at Bordeaux, Clermont-Ferrand, Limoges, Montpellier, and Toulouse). Data were collected from December 2018 to July 2019.

### Study Patients

All adult outpatients and hospitalized patients were eligible to be included in this study if they were diagnosed with RA according to the 2019 ACR/EULAR (American College of Rheumatology/European League Against Rheumatism) classification criteria [10]. Eligible patients were systematically asked to participate in the survey, and if they agreed, they were included in the study. Patients who refused to participate or had language difficulties were excluded from the study. All patients provided written informed consent and agreed to participation. The local ethics committee (number 2018-A01875-50) approved this study.

### Data Collection

Data were collected during a single visit through an anonymous patient self-questionnaire; this included sociodemographic data, medical data, and assessment of the use of eHealth tools (ie, access, support, frequency of use, type of use, and reason for use). The use of eHealth for RA was defined as “to possess an electronic tool” and “to use it to get information or to manage RA.” The rheumatologist in charge of the patient completed an independent medical questionnaire collecting RA characteristics and comorbidities (details on data collected are available in Multimedia Appendix 1).

### Statistical Analysis

Qualitative variables were described as absolute values and percentages. Quantitative variables were described as mean (SD) or median (IQR) values. Demographic characteristics, disease characteristics, disease activity, treatment intake, and comorbidity index were compared between eHealth tools users and nonusers. Additionally, chi-square test or Fisher exact test, as required, was used to compare qualitative variables. Student *t*-test was used to compare quantitative variables with normal distribution and homogeneous variances. Logistic regression, with backward procedure to select variables, was used for multivariate analysis to identify patients’ characteristics that were independently associated with eHealth use for RA. All variables associated with eHealth use for RA with *P*<.1 in univariate analyses were tested in the model. The results are described with odds ratio (OR). Statistical analyses were carried out using STATA software (version 13.1; StataCorp).

## Results

### eHealth Use in RA populations

**Figure 1:**
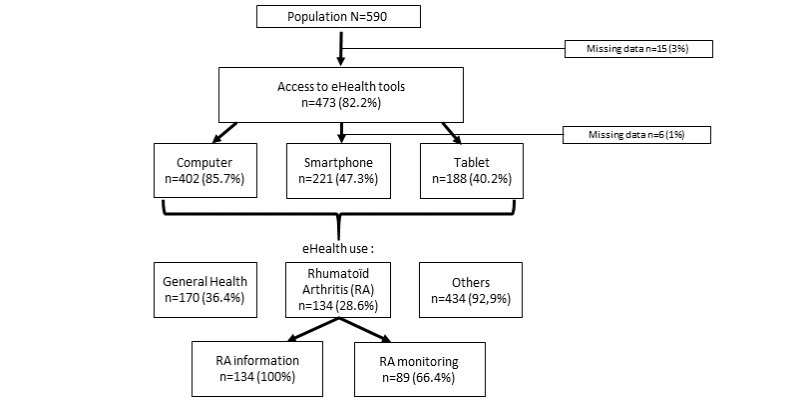
Use of eHealth tools in the study population.

A total of 575 patients completed the self-questionnaire. We found that 82.2% (473/575) of the patients had an eHealth tool, and 28.7% (134/467) of them used it for RA. All of these 134 patients (100%) used the eHealth tool to obtain information about RA, and 89 (66.4%) of them used it to monitor their rheumatism. Figure 1 shows the use of eHealth tools in the study population. Table 1 shows the modalities of use and the frequency of access to eHealth for RA. Most patients used the internet infrequently.

**Table 1:** Frequency and modalities of use of the internet by study patients using eHealth for rheumatoid arthritis.

Modality of use		Frequency of use		
		Often used (>1/month), n (%)	Sometimes used (≤1/month), n (%)	Never used, n (%)
**eHealth content**				
	Rheumatoid arthritis information (n=128)	15 (11.7)	92 (71.8)	21 (16.4)
	Treatment information (n=128)	21 (16.4)	83 (64.8)	24 (18.7)
	Patient forums (n=122)	13 (10.6)	45 (36.8)	64 (52.4)
	Social network (n=123)	16 (13)	16 (13)	91 (73.9)
	Communication with the rheumatologist (n=123)	4 (3.2)	26 (21.1)	93 (75.6)
	Appointment management (n=122)	8 (6.5)	33 (27)	81 (66.3)
**Websites**				
	Generic French websites (eg, Wikipedia), (n=121)	16 (13.2)	55 (45.4)	50 (40.9)
	Rheumatology-specific French websites (eg, rhumato.net, ANDAR, SPILF), (n=123)	28 (22.7)	55 (44.7)	40 (32.5)
	Hospital center websites (n=119)	4 (3.3)	29 (24.3)	86 (72.2)
	Pharmaceutical industry websites (n=120)	3 (2.5)	17 (14.1)	100 (83.3)

### Association Between Patient Characteristics and eHealth Use for RA

Factors such as age, education level, employment, DMARDs, level of comorbidity, membership of a patient association, and participation in a patient education program were found to be associated with the use of eHealth tools for RA (Table 2).

Multivariate analysis showed that membership of a patient association remained independently associated with the use of eHealth tools for RA (OR 5.8, 95% CI 3.0-11.2; *P*<.001), whereas a high level of comorbidity (OR 0.7, 95% CI 0.6-0.8; *P*<.001) and use of biologic DMARDs (OR 0.6, 95% CI 0.4-1.0; *P*<.041) were associated with a lower use of eHealth tools.

**Table 2:** Patient characteristics and factors associated with the use of eHealth tools. Italicized values indicate statistically significant values.

Characteristic			Patients using eHealth for RA^a^ (n=134)	Patients not using eHealth (n=441)	*P* value
**Demographic characteristics**					
		Gender (female), n (%)	110/134 (82)	337/440 (76.5)	.18
		Age (years), mean (SD), (n=569)	58.4 (13.2)	62.8 (12.1)	*<.001*
		BMI (kg/m²), mean (SD), (n=553)	25.2 (5.0)	25.7 (5.1)	.32
	**Living place, n (%)**				.35
		Urban	60/132 (45.4)	174/426 (40.8)	
		Rural	72/132 (54.5)	252/426 (59.1)	
	**Education level, n (%)**				*.006*
		Undergraduate or middle school	53/126 (42)	237/424 (55.8)	
		High school or college	73/126 (57.9)	187/424 (43.3)	
	**Employment status,** **n (%)**				*.006*
		Farmer, artisan, worker	10/132 (7.5)	45/431 (10.4)	
		Senior framework, employed	49/132 (37.1)	97/431 (22.5)	
		Unemployed	19/132 (14.3)	58/431 (13.4)	
		Retired	54/132 (40.9)	231/431 (53.5)	
	**RA characteristics**				
		RA duration (years), mean (SD), (n=551)	15.2 (12.0)	15.8 (10.0)	.56
		RF^b^+, n (%)	101/123 (82.1)	333/421 (79.1)	.46
		ACPA^c^+, n (%)	101/123 (82.1)	339/416 (81.4)	.88
		Erosive, n (%)	73/125 (58.4)	274/423 (64.7)	.19
		DAS28^d^-CRP^e^, mean (SD), (n=505)	2.5 (1.2)	2.6 (1.2)	.84
	**Treatments**				
		Corticosteroids, n (%)	32/128 (25)	92/426 (21.5)	.42
		csDMARDs^f^ monotherapy (MTX^g^ or other csDMARDs), n (%)	44/128 (34.3)	108/425 (25.4)	*.046*
		bDMARDs^h^ (IV^i^, SC^j^), n (%)	75/128 (58.5)	304/425 (71.5)	*.006*
		Association of csDMARDs + bDMARDs, n (%)	49/128 (38.2)	199/425 (46.8)	.09
		Comorbidity (Charlson index), mean (SD), (n=545)	1.8 (1.5)	2.7 (2.0)	*<.001*
	Membership of a patient association, n (%)		29/132 (21.9)	22/432 (5)	*<.001*
	Patient education program, n (%)		47/131 (35.8)	88/428 (20.5)	*<.001*

^a^RA: rheumatoid arthritis.

^b^RF: rheumatoid factor.

^c^ACPA: anticitrullinated protein antibodies.

^d^DAS28: Disease Activity Score-28.

^e^CRP: c-reactive protein.

^f^csDMARDs: conventional synthetic disease-modifying antirheumatic drugs.

^g^MTX: methotrexate.

^h^bDMARDs: biologic disease-modifying antirheumatic drugs.

^i^IV: intravenous.

^j^SC: subcutaneous.

Regarding health applications, 21 (16.5%) and 27 (21.1%) of the 127 patients used ehealth applications (eg, sports, nutrition, diabetes, or cardiovascular) and specific rheumatic applications (Hiboot, J’agis, Arthritis, Sanoia, or myPR), respectively.

A limited group of patients used electronic technology for RA-related follow-up (Excel spreadsheets, 10/125, 8%; mobile app, 9/125, 7.2%; website, 5/125, 4%; paper record, 87/125, 69.6%; and none, 31/125, 24.8%) and medication reminder (electronic diary, 13/125, 10.4%; mobile app, 3/125, 2.4%; clock, 7/125, 5.6%; paper diary, 16/125, 12.8%), whereas 67.2% (84/125) of them used it for no specific reason.

### Patients’ Expectations About eHealth for RA

When investigating patients’ expectations, 225 of 330 patients (68.2%) reported they would use eHealth to manage their RA if it were recommended by a doctor. eHealth device characteristics such as ease of use and security were mentioned by 105 (31.8%) and 69 (20.9%) of the 330 patients, respectively, to increase their adherence to eHealth, and 89 (27%) patients declared they would more likely use eHealth in the case of an RA flare.

## Discussion

To the best of our knowledge, no study thus far has investigated the frequency of eHealth use in a specific population of RA. In this study, we found that 473 of 575 (82.2%) patients had electronic devices and about one-third of those used eHealth specifically for RA to obtain information about the disease and to help with disease management. However, specific use of a mobile app for RA as digital tool to remind themselves about medications or follow-up was rarely reported.

Digital device possession in this RA population was comparable to that in a French population in a previous study [11] and in other studies examining chronic diseases (eg, cancer and cardiovascular diseases), but it was lower than that reported in a diabetes population (55%-84% in available studies) [12-14] perhaps due to the more recent availability of these devices. Moreover, the therapeutic target is not objectively measurable by patients with RA, whereas blood glucose levels can be self-measured on a daily basis by patients with diabetes and consequent therapeutic changes can be decided by the patient after they have completed an educational program. Therapeutic changes for patients with RA, however, are complex decisions made by a physician.

As previously shown in studies examining other chronic diseases [12,14-16], membership of a patient association is strongly associated with eHealth use, which suggests that networking among patients is an effective way to enhance eHealth use.

Closer follow-up (eg, day hospitalizations and consultations) of patients with RA receiving biotherapy may explain the reduced need for eHealth. Similarly, patients with increased comorbidities, who are also older, are more likely to make more frequent medical visits and use eHealth less frequently.

The low frequency of eHealth use for RA could be explained by patients and disease barriers. For example, older patients use eHealth less often, and pain and joint deformations can restrict the use of eHealth devices among these patients. Patients’ habits and disease activity may also affect the usefulness of eHealth (eg, patients in sustained remission might regard the use of eHealth to monitor their disease as futile) [6,17,18].

Other limitations include the lack of information provided by health professionals about the different eHealth tools available and the discrepancy of eHealth use by physicians [19], thus resulting in the lack of promotion of these instruments. Finally, evidence-based medicine [20] and data security [21] are fundamental aspects; however, the utility and data security of the majority of eHealth tools have never been assessed.

Our study has several limitations. First, data were collected with a declarative questionnaire that may not reflect the exact use of eHealth and increase the risk of missing data or false answers. However, the rate of missing data was very low in this study (ie, 3%), and false answers were minimized since the questionnaires were anonymously completed by patients who were informed that their rheumatologists would remain blinded to their answers. The strength of this study was its multicenter design with a systematic inclusion of consecutive patients, which resulted in a representative sample of patients with RA in France.

In the context of the current COVID-19 pandemic where in-person consultations are limited, eHealth, which allows for remote monitoring, remote auto-evaluation of disease activity, and teleconsultation, has gained considerable interest and continues to be developed [22].

In summary, the frequency of eHealth use for RA is low in France, especially in patients with multimorbidities and severe disease and those using biologic DMARDs. Further studies need to be conducted to evaluate the reasons regarding the use and nonuse of eHealth by patients with RA. Furthermore, studies assessing the efficacy of eHealth tools and their impact on patient adherence are necessary before these tools are professionally recommended to patients.

## Appendix

Multimedia Appendix 1 Data collection methods involving an anonymous self-questionnaire for patients and an anonymous and independent medical questionnaire for the rheumatologist in charge.

## Abbreviations

DMARDs: disease-modifying antirheumatic drugs
OR: odds ratio
RA: rheumatoid arthritis

## References

[1] Guillemin F, Saraux A, Guggenbuhl P, Roux CH, Fardellone P, Le Bihan E, Cantagrel A, Chary-Valckenaere I, Euller-Ziegler L, Flipo R-M, Juvin R, Behier J-M, Fautrel B, Masson C, Coste J (2005). Prevalence of rheumatoid arthritis in France: 2001. Ann Rheum Dis.

[2] Eysenbach G (2001). What is e-health?. J Med Internet Res.

[3] Liu W, Huang C, Wang C, Lee K, Lin S, Kuo H (2011). A mobile telephone-based interactive self-care system improves asthma control. Eur Respir J.

[4] Charpentier G, Benhamou P, Dardari D, Clergeot A, Franc S, Schaepelynck-Belicar P, Catargi B, Melki V, Chaillous L, Farret A, Bosson J, Penfornis A, TeleDiab Study Group (2011). The Diabeo software enabling individualized insulin dose adjustments combined with telemedicine support improves HbA1c in poorly controlled type 1 diabetic patients: a 6-month, randomized, open-label, parallel-group, multicenter trial (TeleDiab 1 Study). Diabetes Care.

[5] Gandapur Y, Kianoush S, Kelli HM, Misra S, Urrea B, Blaha MJ, Graham G, Marvel FA, Martin SS (2016). The role of mHealth for improving medication adherence in patients with cardiovascular disease: a systematic review. Eur Heart J Qual Care Clin Outcomes.

[6] Gossec L, Cantagrel A, Soubrier M, Berthelot J, Joubert J, Combe B, Czarlewski W, Wendling D, Dernis E, Grange L, Beauvais C, Perdriger A, Nataf H, Dougados M, Servy H (2018). An e-health interactive self-assessment website (Sanoia) in rheumatoid arthritis. A 12-month randomized controlled trial in 320 patients. Joint Bone Spine.

[7] Salaffi F, Carotti M, Ciapetti A, Di Carlo M, Gasparini S, Farah S, Gutierrez M (2016). Effectiveness of a telemonitoring intensive strategy in early rheumatoid arthritis: comparison with the conventional management approach. BMC Musculoskelet Disord.

[8] Lorig KR, Ritter PL, Laurent DD, Plant K (2008). The internet-based arthritis self-management program: a one-year randomized trial for patients with arthritis or fibromyalgia. Arthritis Rheum.

[9] El Miedany Y, El Gaafary M, Youssef S, Bahlas S, Almedany S, Ahmed I, Palmer D (2016). Toward electronic health recording: evaluation of electronic patient-reported outcome measures system for remote monitoring of early rheumatoid arthritis. J Rheumatol.

[10] Aletaha D, Neogi T, Silman A, Funovits J, Felson D, Bingham C, Birnbaum NS, Burmester GR, Bykerk VO, Cohen MD, Combe B, Costenbader KH, Dougados M, Emery Paul, Ferraccioli G, Hazes JMW, Hobbs K, Huizinga TWJ, Kavanaugh A, Kay J, Kvien TK, Laing T, Mease P, Ménard HA, Moreland LW, Naden RL, Pincus T, Smolen JS, Stanislawska-Biernat E, Symmons D, Tak PP, Upchurch KS, Vencovský J, Wolfe F, Hawker G (2010). 2010 Rheumatoid arthritis classification criteria: an American College of Rheumatology/European League Against Rheumatism collaborative initiative. Arthritis Rheum.

[11] (2018). Baromètre du numérique 2018. CRÉDOC.

[12] Eysenbach G (2003). The impact of the internet on cancer outcomes. CA Cancer J Clin.

[13] Hansen AH, Broz J, Claudi T, Årsand E (2018). Relations between the use of electronic health and the use of general practitioner and somatic specialist visits in patients with type 1 diabetes: cross-sectional study. J Med Internet Res.

[14] Zhang Y, Lauche R, Sibbritt D, Olaniran B, Cook R, Adams J (2017). Comparison of health information technology use between American adults with and without chronic health conditions: findings from the National Health Interview Survey 2012. J Med Internet Res.

[15] Jamal A, Khan SA, AlHumud A, Al-Duhyyim A, Alrashed M, Bin Shabr F, Alteraif A, Almuziri A, Househ M, Qureshi R (2015). Association of online health information-seeking behavior and self-care activities among type 2 diabetic patients in Saudi Arabia. J Med Internet Res.

[16] Mouelhi Y, Alessandrini M, Pauly V, Dussol B, Gentile S (2017). Internet and social network users' profiles in renal transplant recipients in France. BMC Nephrol.

[17] Chrischilles E, Hourcade J, Doucette W, Eichmann D, Gryzlak B, Lorentzen R, Wright K, Letuchy E, Mueller M, Farris K, Levy B (2014). Personal health records: a randomized trial of effects on elder medication safety. J Am Med Inform Assoc.

[18] Mollard E, Michaud K (2018). A mobile app with optical imaging for the self-management of hand rheumatoid arthritis: pilot study. JMIR Mhealth Uhealth.

[19] Vogt F, Seidl F, Santarpino G, van Griensven M, Emmert M, Edenharter G, Pförringer D (2018). Healthcare IT utilization and penetration among physicians: novel IT solutions in healthcare - use and acceptance in hospitals. Eur Surg Res.

[20] Knitza J, Tascilar K, Messner E, Meyer M, Vossen D, Pulla A, Bosch P, Kittler J, Kleyer A, Sewerin P, Mucke J, Haase I, Simon D, Krusche M (2019). German mobile apps in rheumatology: review and analysis using the Mobile Application Rating Scale (MARS). JMIR Mhealth Uhealth.

[21] Blenner SR, Köllmer M, Rouse AJ, Daneshvar N, Williams C, Andrews LB (2016). Privacy policies of Android diabetes apps and sharing of health information. JAMA.

[22] Ohannessian R, Duong TA, Odone A (2020). Global telemedicine implementation and integration within health systems to fight the COVID-19 pandemic: a call to action. JMIR Public Health Surveill.

