# Supplementary material for: Use of eHealth by Patients With Rheumatoid Arthritis: Observational, Cross-sectional, Multicenter Study
Source: J Med Internet Res. 2021 Jan 29;23(1):e19998. doi: 10.2196/19998 (PMC7880811; doi:10.2196/19998)
Supplement: Multimedia Appendix 1 [file jmir_v23i1e19998_app1.docx]

**Supplementary data: patients and methods**

Data collection

The anonymous self-questionnaire completed by the patients included:

- sociodemographic questions (sex, date of birth, weight, high, place, employment, level of education);
- general medical questions and RA-specific questions (smoking, alcohol intake, membership of patient association group, patient education program participation);
- use of connected devices for eHealth (access and frequency of use of the Internet and eHealth tools: Internet, computer, tablet, smartphone, other connected devices; reasons of use; knowledge and use of RA-specific websites and apps (such as websites providing RA information, RA treatment, forum of patients, social network, communication with the rheumatologist, appointment management, general French language websites, rheumatoid specific websites, hospital center websites and pharmaceutical laboratory websites); RA follow up (Excel® spreadsheets, mobile app, website, paper record, nothing), medication recording (electronic diary, mobile app, clock, paper diary or nothing); patient’s expectation of digital devices (tool recommended by a doctor, a friend, a RA patient or a member of association, the safety of the device, data security, RA activity) ;

More specifically, we asked patients about the use of specific rheumatic mobile apps. We have selected all the French language mobile apps available on Android or iOS at the time of the study:

- Hiboot®: developed by the French Society of Rheumatology. Available since May 2017 on Android and iOS. It is intended as a tool to help improve the quality of life. It provides access to information on inflammatory rheumatism, recommendations, monitoring of the disease with the collection of PROs (fatigue, pain ...), and helps to recall and monitor drug intake (verification of contraindications before taking), through an interactive interface. (more information on: https://[www.hiboot.fr](about:blank)/)
- J'agis®: developed by Pfizer Pharm. Available on Android and iOS. It provides information about the disease, follow up with a logbook (including symptoms, location flare-up, biological assessments), reminders of appointments or medications. (more information on: https://[www.pfizer.fr](about:blank)/)
- ArthritisID®: developed by Canadian 1pp. It helps to classify symptoms of arthritis as chronic rheumatism. (more information on: [https://www.arthritisresearch.ca/](about:blank))
- Ma PR®: developed by Roche-Chugaï App. It helps to locate arthritis, follow up with a logbook, to remind of medication doses and appointments, and to speak to the rheumatologist. (more informations on: https://[www.roche.fr](about:blank)/)

The rheumatologist completed an anonymous and independent medical questionnaire that gathered the following information:

- disease characteristics (RA duration, Rheumatoid factor and ACPA presence), disease activity (level of CRP, erythrocyte sedimentation rate (ESR), number of tender joints, number of swollen joints, patient global health evaluation on the Visual Analogue Scale (VAS) from 0 to 100, disease activity score (DAS28-CRP and DAS28-ESR)) (1), previous RA treatments (current treatments (csDMARD, bDMARD, corticoids), route of administration, current drugs count) ;
- comorbidity score using the Charlson comorbidity index (2). Charlson comorbidity index defines 19 comorbidities. It predicts 10-year survival in patients with multiple comorbidities.
